# Research trends and hotspots for frontotemporal dementia from 2000 to 2022: a bibliometric analysis

**DOI:** 10.3389/fneur.2024.1399600

**Published:** 2024-07-17

**Authors:** Xinxin Chen, Yin Chen, Biyu Ni, Cheng Huang

**Affiliations:** ^1^Department of Rehabilitation Medicine, West China Hospital, Sichuan University, Chengdu, Sichuan, China; ^2^Department of Rehabilitation Medicine, The First Affiliated Hospital of Sun Yat-sen University, Guangzhou, China; ^3^Key Laboratory of Rehabilitation Medicine in Sichuan Province, West China Hospital, Sichuan University, Chengdu, Sichuan, China

**Keywords:** bibliometric analysis, frontotemporal dementia, Alzheimer’s disease, Citespace, Vosviewer

## Abstract

**Background:**

Frontotemporal dementia (FTD) is a neurodegenerative disease with clinical, pathological, and genetic heterogeneity. FTD is receiving increasing attention because it is the second leading cause of early-onset dementia after Alzheimer’s disease. This study aimed to analyse the research trends and hotspots of FTD from 2000 to 2022 using bibliometrics.

**Methods:**

Papers related to FTD from 2000 to 2020 were systematically searched through the Web of Science Core Collection (WOSCC). Citespace and Vosviewer software were used to visually analyse the retrieved data of countries/regions, institutions, journals, authors, references, and keywords. Microsoft Excel was used to generate the annual publications and growth trends.

**Results:**

There were 10,227 papers included in the bibliometric analysis. The annual publication output on FTD has increased significantly from 2000 to 2022, with papers published in 934 academic journals and 87 countries/regions. The Journal of Alzheimer’s Disease was the most popular, with 488 papers about FTD. The most productive countries/regions, institutions, and authors are the United States (*n* = 4,037), the University of California San Francisco (*n* = 687), and Miller, Bruce L. (*n* = 427), respectively. The article by Katya Rascovsky and her colleagues published on Brain in 2011 was the most cocited paper, with 625 citations. The research hotspots in this field were the clinical diagnostic criteria, subdivision, and pathological mechanism of FTD, such as tau protein, chromosome 17, progranulin, TDP-43, and C9orf72.

**Conclusion:**

The future research direction is based on biomarkers and pathological mechanisms to diagnose and differential diagnose FTD from the aspects of behavior, neuropathology, neuroimaging, and serum markers.

## 1 Introduction

Frontotemporal dementia (FTD) is a neurodegenerative clinical syndrome characterized by progressive changes in executive function, behavior, language, or motor function (1). The neuroanatomical features of FTD are correlated with impairment and neuronal loss in the frontal and temporal lobes and now include more extensive cortical, subcortical, brainstem, and cerebellar involvement (2). FTD is the third leading cause of late-onset dementia (age ≥ 65 years) after Alzheimer’s disease (AD) and Lewy body dementia and the second leading cause of early-onset dementia (age < 65 years) after AD (1, 3). FTD accounts for 10–20% of all dementia cases (4). One meta-analysis suggested that the prevalence of FTD ranged from 3 to 26% (5). However, the disease is easily missed and misdiagnosed, which may underestimate the true prevalence (1). Clinically, FTD can be divided into the behavioral variant of FTD (bvFTD) and primary progressive aphasia (PPA) (6). PPA includes two subtypes, namely, nonfluent variant PPA (nfvPPA) and semantic variant PPA (svPPA). In addition, FTD may co-exist with related motor neurone disease, Parkinson’s disease, corticobasal syndrome, or progressive supronuclear palsy (PSP), which may be special subtypes of FTD. PSP is characterized by vertical supranuclear ophthalmoplegia, extrapyramidal muscle rigidity, gait ataxia, and mild dementia (7). In addition to pure forms of PSP, PSP pathology can be found in patients with Parkinson’s disease and frontotemporal dementia, leading to possible misdiagnosis of PSP (8). The two main aggregated proteins in the brains of patients with FTD are tau and TDP-43, while a small number of patients aggregate in fused-in-sarcoma proteins (9). Approximately 40% of people with FTD have a family history (10), and one-third of FTD is inherited by autosomal dominant mutations in three genes: progranulin (GRN), microtubule-associated protein tau (MAPT) and chromosome 9 open reading frame 72 (C9orf72) (11). Several FTD syndromes (bvFTD, svPPA, and nfvPPA) are pathologically clinically and radiologically heterogeneous (12). With the development of neuroimaging technology, studies have revealed numerous biomarkers for various syndromes of FTD, thus narrowing the differential diagnosis and improving diagnostic accuracy (2, 12, 13). The two techniques most commonly used to evaluate FTD in clinical and research settings are structural magnetic resonance imaging (MRI) and positron emission tomography. Currently, there are no FDA-approved treatments for FTD (4). FTD treatment is limited to symptom control, and most compounds used to treat AD-type dementia are ineffective against FTD (9). Therapies targeting genetic forms of FTD have shown significant progress, and noninvasive neuroregulatory techniques have shown potential in alleviating symptoms and enhancing cognition of patients with FTD (14). FTD is of increasing concern, as it accounts for up to 10% of middle-age-onset dementia and imposes a social, financial and emotional burden on patients and caregivers (15). There has been a significant increase in publications on FTD research, including genetics, pathology, neuroimaging, and therapeutic interventions.

Bibliometric analysis is a quantitative method to explore the development trends and research hotspots of certain scientific fields (16). General reviews were studied by individuals through literature summary and extraction and thus cannot visually provide the temporal and spatial distribution of countries, institutions, authors, and journals (17). A well-organized bibliometric analysis can save researchers and clinicians time by providing a clearer overview, cutting-edge hotspots, and trends in a specific field (18). In recent years, bibliometrics has been widely used to analyse neurodegenerative diseases such as AD (19, 20), Parkinson’s disease (21), multiple sclerosis (22), and cerebral amyloid angiopathy (23). To our knowledge, only Guido et al. (24) included 1,436 articles and reviews to conduct a bibliometric analysis of FTD in 2015. FTD has attracted increasing attention, with a growing number of publications which include improvements in clinical, genetic and molecular characteristics, advances in plasma and cerebrospinal fluid biomarkers, and innovations in structural and functional imaging, providing new insights into FTD (25–31). It is necessary to update a bibliometric analysis to better show the current research status, research hotspots and development trends. This study aimed to comprehensively analyse the global research status, research hotspots and trends of FTD from 2000 to 2022 by conducting a bibliometric analysis. These findings may help quantify the characteristics of countries, institutions, journals and authors, as well as analyse citations, keywords and research trends.

## 2 Materials and methods

### 2.1 Data source

**Figure 1:**
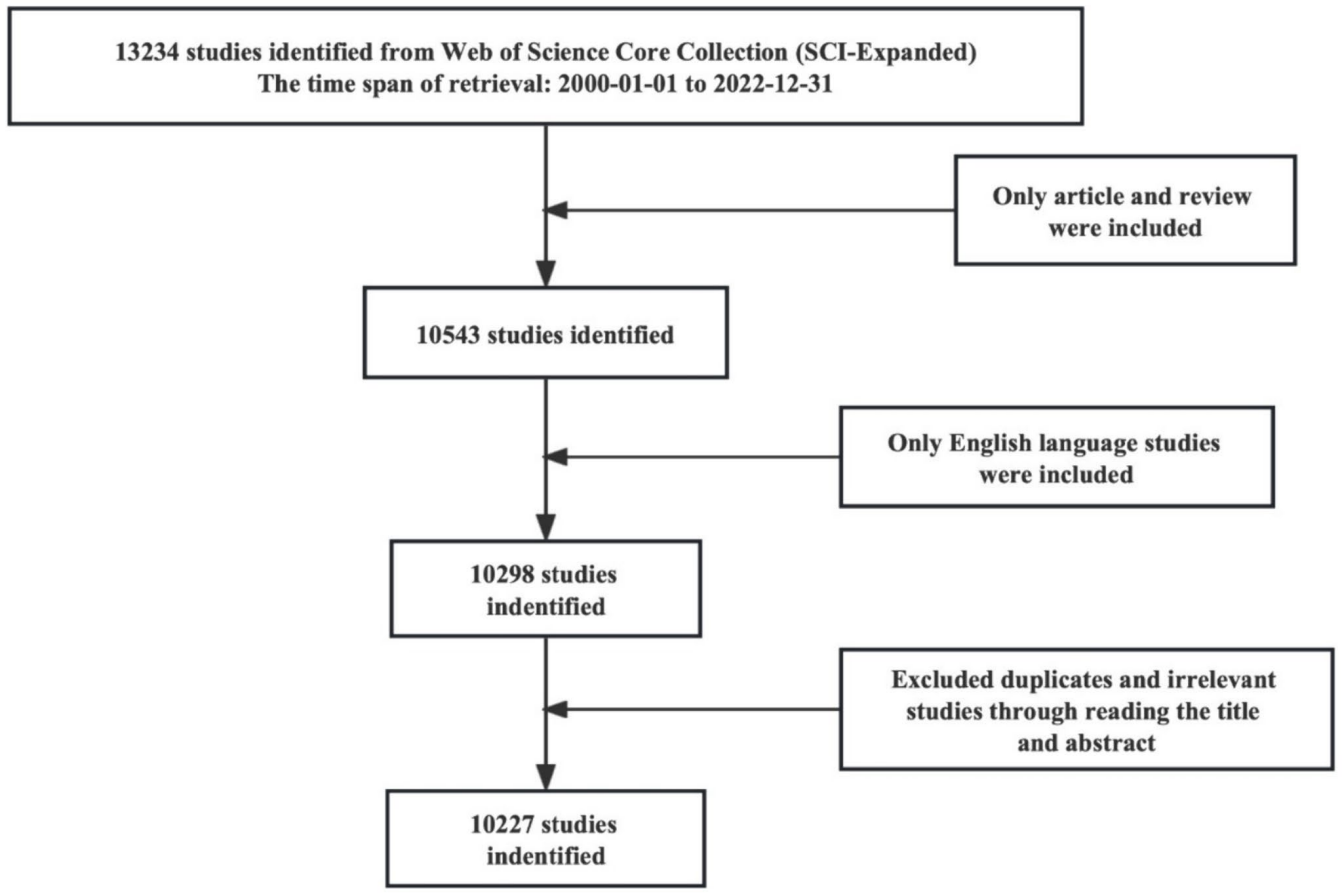
Flow chart of the literature screen.

Full records of publications related to frontotemporal dementia were retrieved and downloaded from the Science Citation Index Expanded (SCI-Expanded) of the Web of Science Core Collection database (WoSCC) on July 5, 2023. The search strategy is shown in Supplementary Appendix A, and the time span was set from January 1, 2000, to December 31, 2022. The type of publication was limited to articles and review articles, and the publication language was confined to English. The retrieved data were independently screened by XXC and CH to confirm the relationship with frontotemporal dementia, and any controversy was resolved by negotiation. Duplicate articles were excluded. Out of the 13,234 articles initially identified, 10,227 were included for further bibliometric analysis. The retrieval process is shown in Figure 1.

### 2.2 Bibliometric analysis

**Figure 2:**
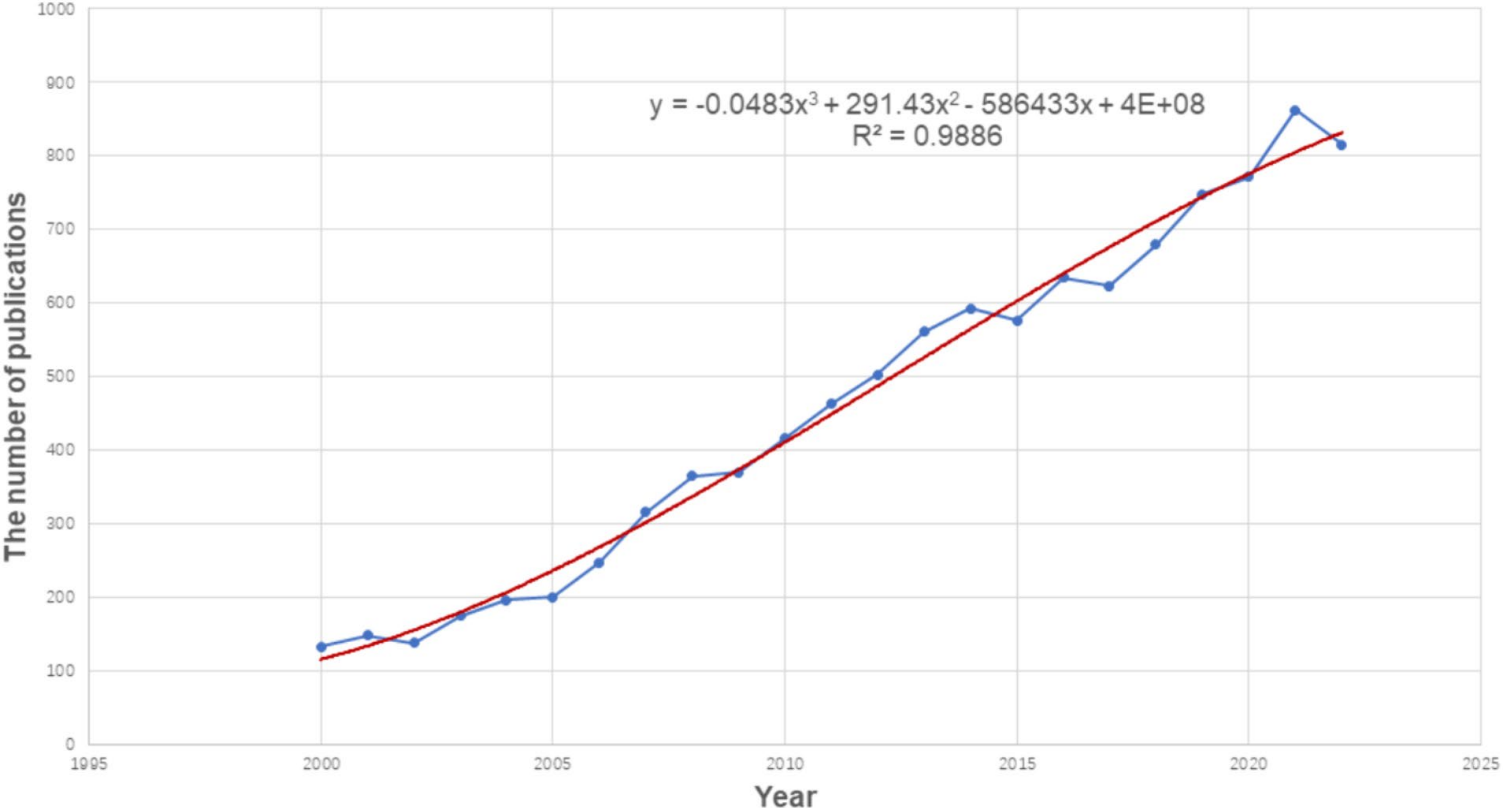
Growth trend of the publication output from 2000 to 2022 on frontotemporal dementia.

The general information for each paper obtained from WoSCC included the number of publications, country, institution, author, title, abstract, keywords, publication year, reference, and citation. This study analyzed several characteristics of publications, including annual publications and growth trends, countries, institutions, journals, authors, cocited authors, cocited references and keywords. All data were downloaded in the plain text format from WoS and then imported to Citespace (version 6.2. R4, 64-bit) and Vosviewer (Version 1.6.19) for further bibliometric analysis. Microsoft Excel 2019 was used to generate the annual publications and growth trends (Figure 2). In the visual map established by Citespace and Vosviewer, each node represents an element, such as a country, institution, journal, or author. The larger the node size is, the greater the number of reflections, and the larger the link between the nodes is, the stronger the degree of collaboration (32).

## 3 Results

### 3.1 Publication output analysis

From 2000 to 2022, the annual publication output increased significantly, as shown in Figure 2, indicating that researchers are paying increasing attention to FTD each year. Since 2012, the number of annual publications has exceeded 500 for 10 consecutive years, and up to 815 by 2022. Linear regression analysis showed that the annual publication volume was positively correlated with the publication year (*R*^2^ = 0.989, *p* < 0.001) (Figure 2). Therefore, we speculate that the annual number of articles in 2023 is expected to reach 900. The number of articles published each year increased year by year, which shows that FTD is an active research field and has received extensive attention from scholars.

### 3.2 Analysis of countries/regions and institutions

**Figure 3:**
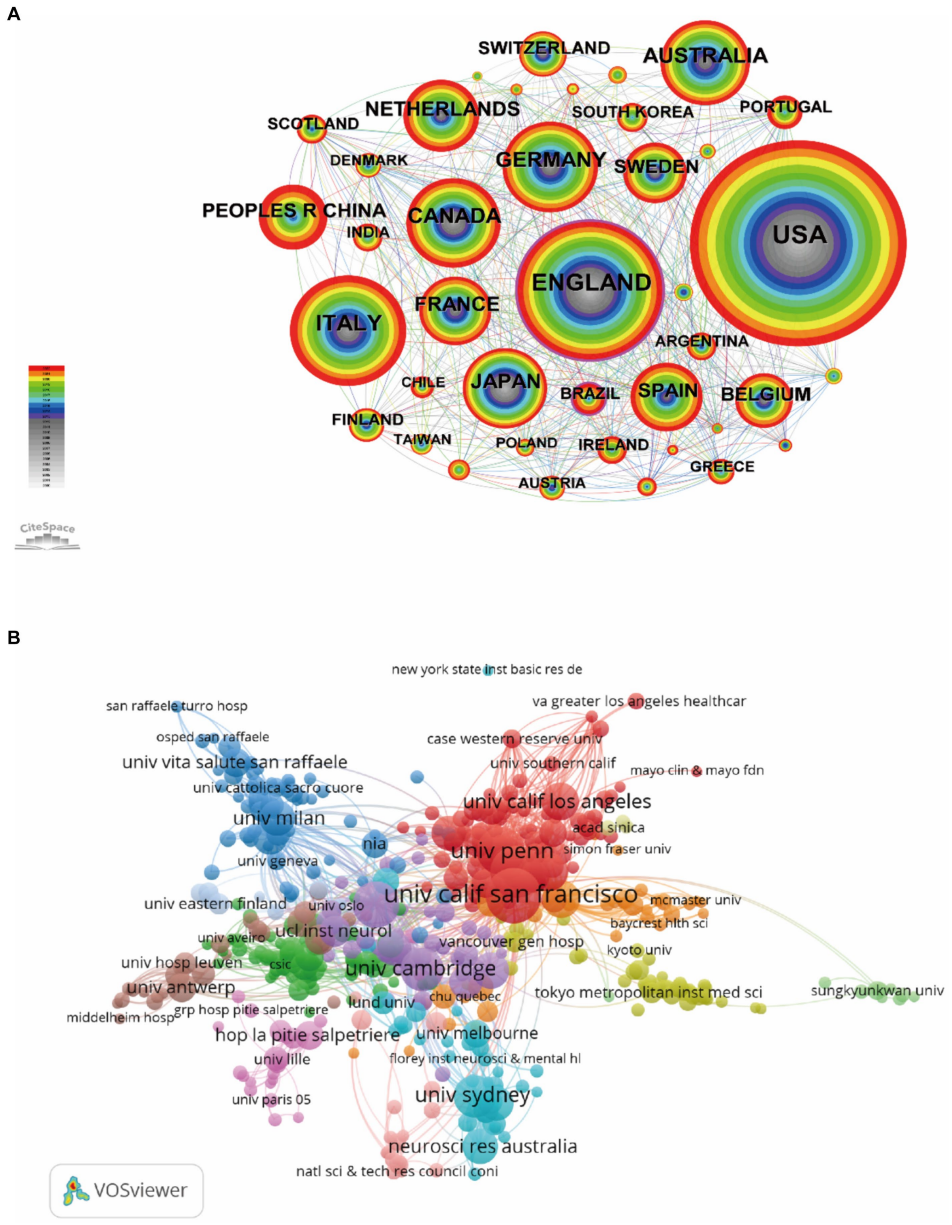
Network map of collaborating countries/regions **(A)** and institutions **(B)**. Each node represents one country/region or institution, the size of the circle represents the number of publications, and the link strength of the connections between two nodes represents the closeness of cooperation.

Analysis of countries/regions and institutions can identify objects with high scientific productivity and influence and present their cooperation in the field. The retrieved publications came from 87 countries/regions. The top 10 countries in terms of number of publications, citations, average citations, and centrality are shown in Table 1. From 2000 to 2022, the United States produced the most publications (*n* = 4,037), followed by England (*n* = 1,872), Italy (*n* = 1,271), Germany (*n* = 906), and Canada (*n* = 884). The top three countries in terms of literature citations and average citations are the United States, England and Canada. Among all countries, the country with the highest centrality is England (0.20). Table 1 also shows the top 10 most productive institutions. The institution with the largest publications is the University of California San Francisco (*n* = 687), followed by Mayo Clinic (*n* = 584), University of Pennsylvania (*n* = 474), University College London (*n* = 455), and University of Cambridge (*n* = 363). Figure 3 illustrates the network map of collaborating countries/regions and institutions. According to the total link strength, we found that the United States has close and extensive partnerships in research with other countries, followed by England, Germany, Italy, Canada, and the Netherlands. Links between the University of California San Francisco, Mayo Clinic, University College London, University of Toronto, University of Pennsylvania, and other institutions indicate close collaboration.

**Table 1:** The top 10 most productive countries/regions and institutions.

Rank	Country/Region	Publications	Citations	Average citation/publication	Centrality	Institution	Publications	Centrality
1	United States	4,037	287,223	71.15	0.08	University of California, San Francisco	687	0.01
2	England	1872	141,691	75.69	0.20	Mayo clinic	584	0.02
3	Italy	1,271	62,168	48.91	0.02	University of Pennsylvania	474	0.04
4	Germany	906	63,940	70.57	0.08	University College London	455	0.01
5	Canada	884	65,439	74.03	0.07	University of Cambridge	363	0.05
6	Australia	842	47,677	56.62	0.02	University of Sydney	336	0.00
7	Japan	717	31,820	44.38	0.07	Northwestern University	274	0.02
8	Netherlands	600	38,255	63.76	0.04	University of Toronto	270	0.05
9	France	561	34,579	61.64	0.09	University of California, Los Angeles	255	0.01
10	Spain	545	21,152	38.81	0.07	University of Manchester	252	0.03

### 3.3 Analysis of published journals

**Figure 4:**
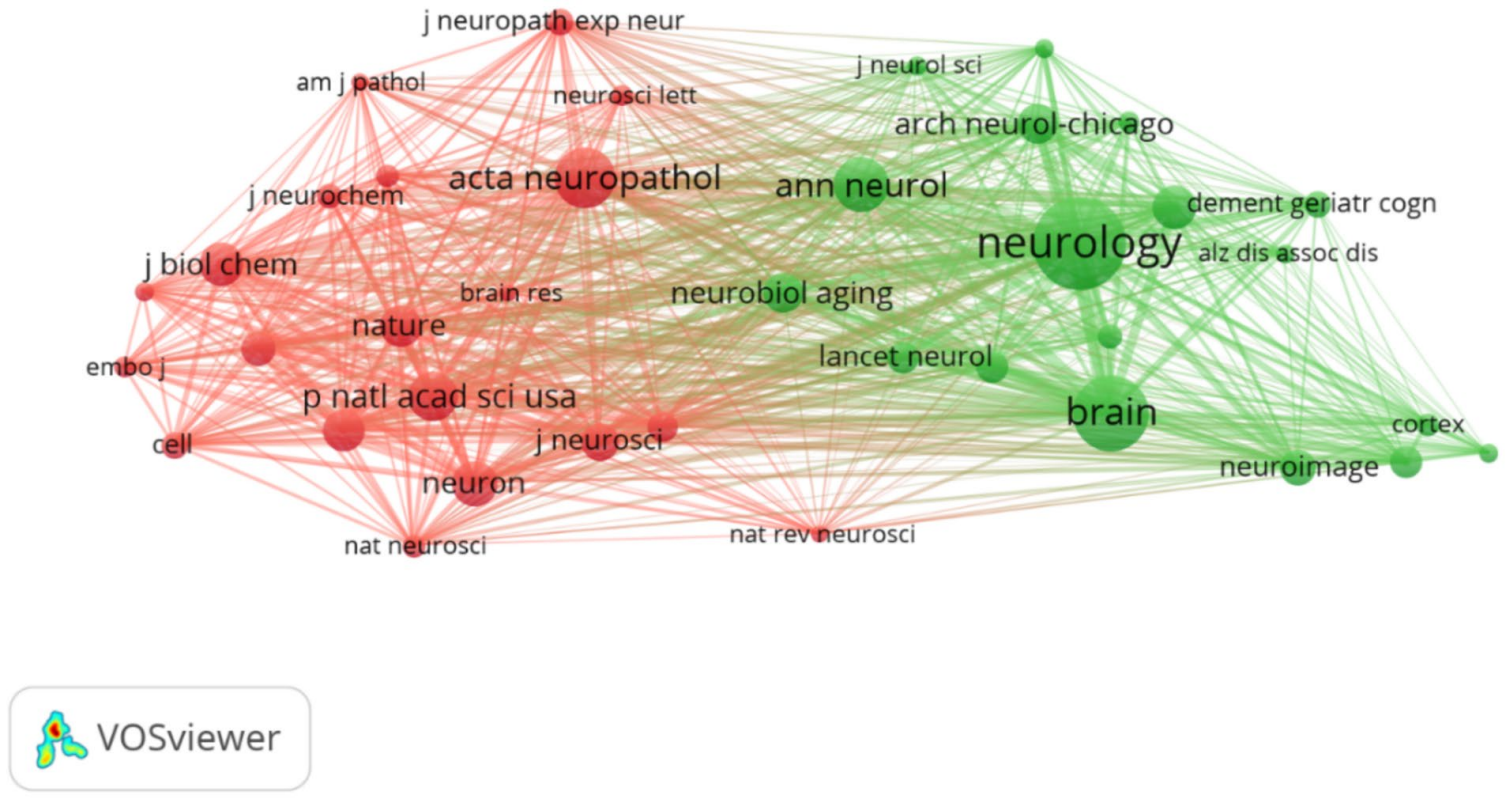
Network map of cocited journals. Each node represents one journal, the size of the circle represents the cocitation counts, and the link connecting the two circles represents the cocited relationship between journals.

Journal analysis can provide a useful reference for publishing research results. A total of 934 different journals published articles on FTD research. Table 2 shows the top 10 journals in terms of publication volume. The Journal of Alzheimer’s Disease (*n* = 488, IF = 4.0) was the most productive journal for publishing FTD-related articles, followed by Neurobiology of Aging (*n* = 344, IF = 4.2), Neurology (*n* = 333, IF = 9.9), Brain (*n* = 302, IF = 14.5), and Acta Neuropathologica (*n* = 275, IF = 12.7). Among the top 10 journals, Brain not only has the greatest number of total citations (*n* = 40,579) but also has the highest impact factor (14.5) and average citations per paper (134.37). Its h-index is 365. It is a journal that provides researchers and clinicians with the best original contributions in the field of neurology, and its continuously published papers have become classics in the field. In the top 10 journals, Neurology has the highest H-index of 396. Journal cocitation analysis can analyse the correlation and similarity between two articles (33). The more cocitations of a journal, the greater the influence of the journal in a specific research field. As shown in Figure 4, Vosviewer identified the top three cocited journals, which were Neurology (38,202), Brain (27,717) and Acta neuropathologica (19,248).

**Table 2:** The top 10 journals that published articles in frontotemporal dementia.

Rank	Journal	Publications	IF (2023)	JCR (2023)	Citations	Average citation/publication	H-index
1	Journal of Alzheimer’s Disease	488	4.0	Q3	11,085	22.72	163
2	Neurobiology of Aging	344	4.2	Q2	10,764	31.29	205
3	Neurology	333	9.9	Q1	37,106	111.43	396
4	Brain	302	14.5	Q1	40,579	134.37	365
5	Acta Neuropathologica	275	12.7	Q1	22,802	82.92	200
6	Dementia and Geriatric Cognitive Disorders	208	2.4	Q4	8,125	39.06	120
7	Journal of Neurology, Neurosurgery and Psychiatry	177	11.0	Q1	10,961	61.93	225
8	Neuropsychologia	167	2.6	Q3	8,517	51.00	221
9	Cortex	153	3.6	Q2	4,383	28.65	131
10	Neurocase	152	0.8	Q4	3,161	20.80	69

### 3.4 Analysis of authors and cocited authors

**Figure 5:**
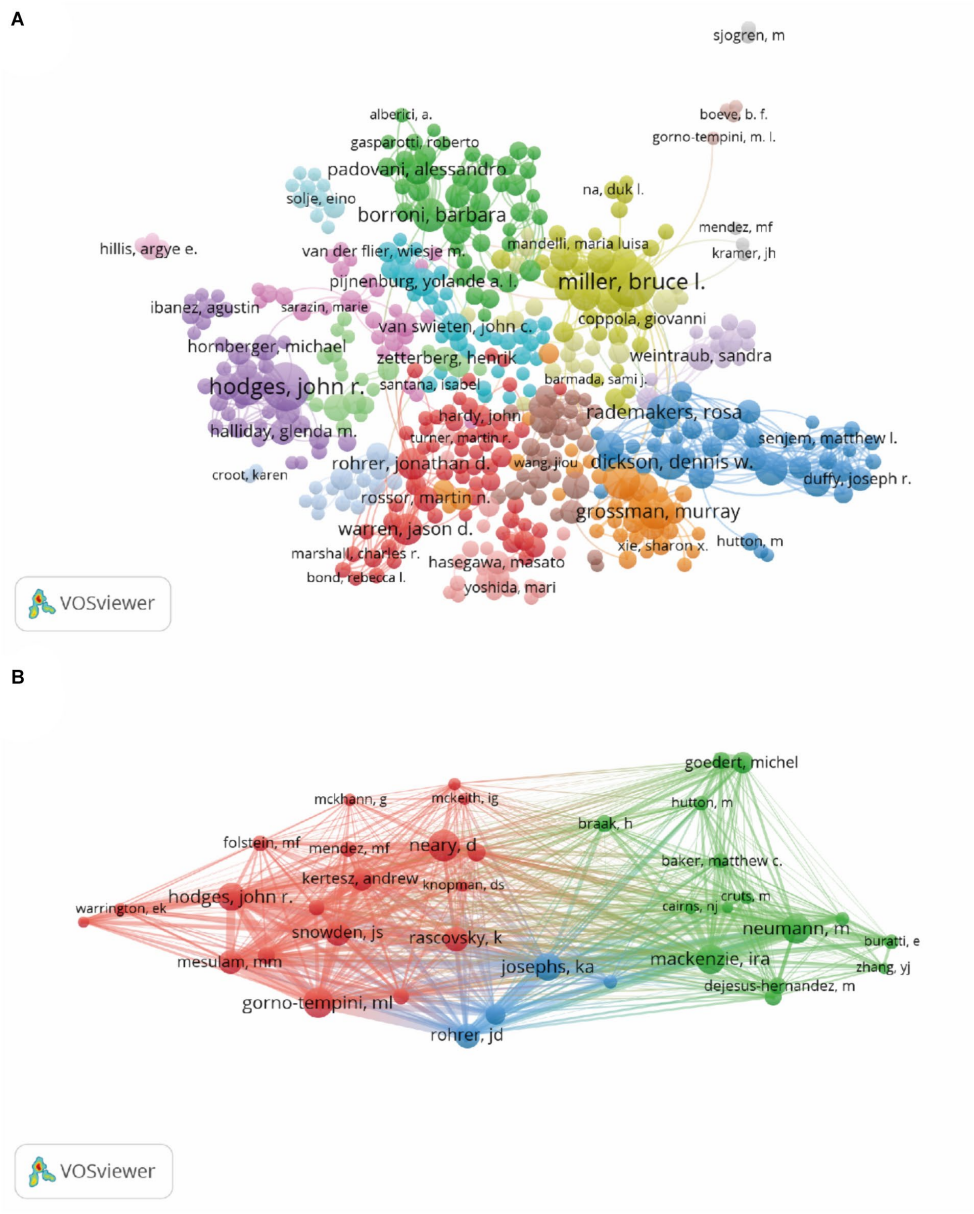
Network map of authors **(A)** and cocited authors **(B)**. Each node represents one author, the size of the circle represents the number of publications **(A)** or cocitation counts **(B)**, and the link connecting the two circles represents the co-occurrence **(A)** or cocitation **(B)** relationship between authors.

The published papers were contributed by 33,909 authors. The top 10 authors and cocited authors that contributed papers on FTD research are shown in Table 3. The most productive authors are Miller, Bruce L. (*n* = 427), followed by Hodges, John R. (*n* = 371), Grossman, Murray (*n* = 200), Dickson, Dennis W. (*n* = 195), and Piguet, Olivier (*n* = 194). Miller, Bruce L. (40,824 citations) had the highest number of citations among all the authors, who focused on FTD, dementia, pathology, Alzheimer’s disease and neuroscience. His body of work in FTD is particularly relevant to semantic dementia. Trojanowski, John Q. had a significantly higher number of citations per item (138.49) than the other authors. Neary, David (3,254 citations) was the most cocited author, followed by Gorno-tempini, ML (3,107 citations), Mackenzie, Ira (3,027 citations), Neumann, M (3,015 citations), and Hodges, John R. (2,862 citations). In frontotemporal lobar degeneration, Neary, David works on issues such as genetics, which are connected to tau protein and charged multivesicular body protein 2B. Figure 5 shows the network maps of authors and cocited authors.

**Table 3:** The top 10 authors and cocited authors that contributed papers in frontotemporal dementia.

Rank	Author	Publications	Citations	Average citation/publication	Cocited authors	Cocitation counts
1	Miller, Bruce L.	427	40,824	95.61	Neary, David	3,254
2	Hodges, John R.	371	32,484	87.56	Gorno-tempini, ML	3,107
3	Grossman, Murray	200	19,137	95.69	Mackenzie, Ira	3,027
4	Dickson, Dennis W.	195	19,281	98.88	Neumann, M	3,015
5	Piguet, Olivier	194	7,142	36.81	Hodges, John R.	2,862
6	Trojanowski, John Q.	193	26,729	138.49	Josephs, Keith A.	2,850
7	Borroni, Barbara	158	5,489	34.74	Snowden, JS	2,439
8	Rademakers, Rosa	152	13,632	89.68	Rascovsky, K	2,337
9	Josephs, Keith A.	145	8,858	61.09	Rohrer, JD	2,327
10	Rohrer, Jonathan D.	134	7,481	55.83	Mesulam, MM	2,216

### 3.5 Analysis of cocited references

**Figure 6:**
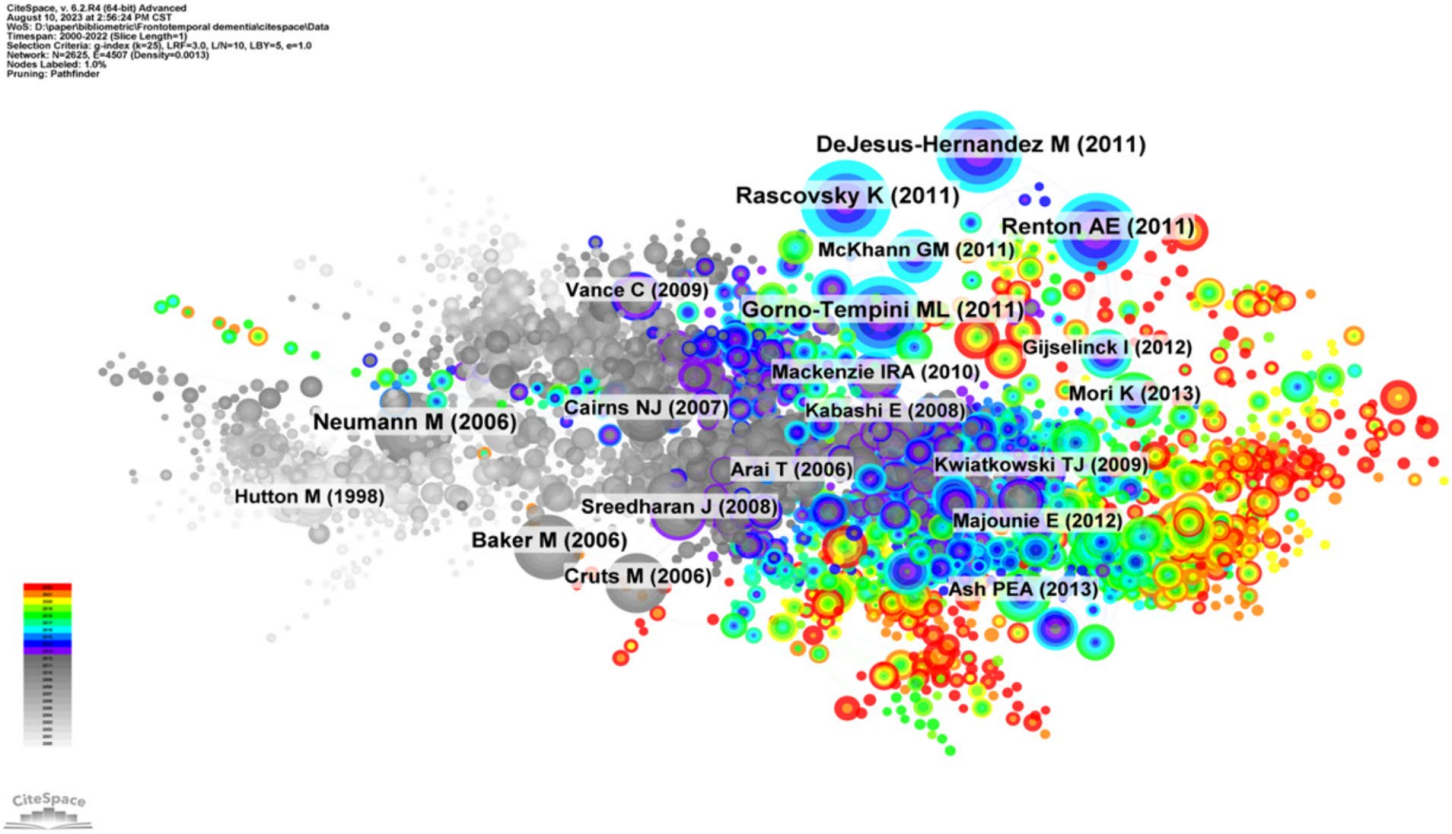
Visualization of the cocited references network. Each node represents one reference, the size of the circle represents the cocitation counts, and the link connecting the two circles represents the cocited relationship between references.

Highly cited articles may lay the foundation for a certain field and often have important reference value (34). Figure 6 shows the network map of cocited references, and Table 4 shows the top 10 cocited references related to frontotemporal dementia. These papers focused on the diagnostic criteria and genetic research topics of FTD. The article by Rascovsky Katya and his colleagues published on *Brain* in 2011 (35) was the most cocited paper, with 625 citations. The authors indicated that the proposed International Behavioral Variant FTD Criteria Consortium criteria provided a sensitive criterion for bvFTD diagnosis, allowing for early identification of the syndrome when disease-modifying therapies are expected to be most effective.

**Table 4:** The top 10 cocited references related to frontotemporal dementia.

Rank	Cocitation counts	Cited-references	Representative author (publication year)	Journal
1	625	Sensitivity of revised diagnostic criteria for the behavioral variant of frontotemporal dementia	Rascovsky Katya (2011)	Brain
2	568	Expanded GGGGCC hexanucleotide repeat in noncoding region of C9ORF72 causes chromosome 9p-linked FTD and ALS	DeJesus-Hernandez Mariely (2011)	Neuron
3	554	A hexanucleotide repeat expansion in C9ORF72 is the cause of chromosome 9p21-linked ALS-FTD	Renton Alan E (2011)	Neuron
4	549	Classification of primary progressive aphasia and its variants	Gorno-Tempini ML (2011)	Neurology
5	468	Ubiquitinated TDP-43 in frontotemporal lobar degeneration and amyotrophic lateral sclerosis	Neumann Manuela (2006)	Science
6	360	Mutations in progranulin cause tau-negative frontotemporal dementia linked to chromosome 17	Baker Matt (2006)	Nature
7	308	Null mutations in progranulin cause ubiquitin-positive frontotemporal dementia linked to chromosome 17q21	Cruts Marc (2006)	Nature
8	266	The C9orf72 GGGGCC repeat is translated into aggregating dipeptide-repeat proteins in FTLD/ALS	Mori Kohji (2013)	Science
9	265	Neuropathologic diagnostic and nosologic criteria for frontotemporal lobar degeneration: consensus of the Consortium for Frontotemporal Lobar Degeneration	Cairns Nigel J (2007)	Acta Neuropathologica
10	256	TDP-43 mutations in familial and sporadic amyotrophic lateral sclerosis	Sreedharan Jemeen (2008)	Science

**Figure 7:**
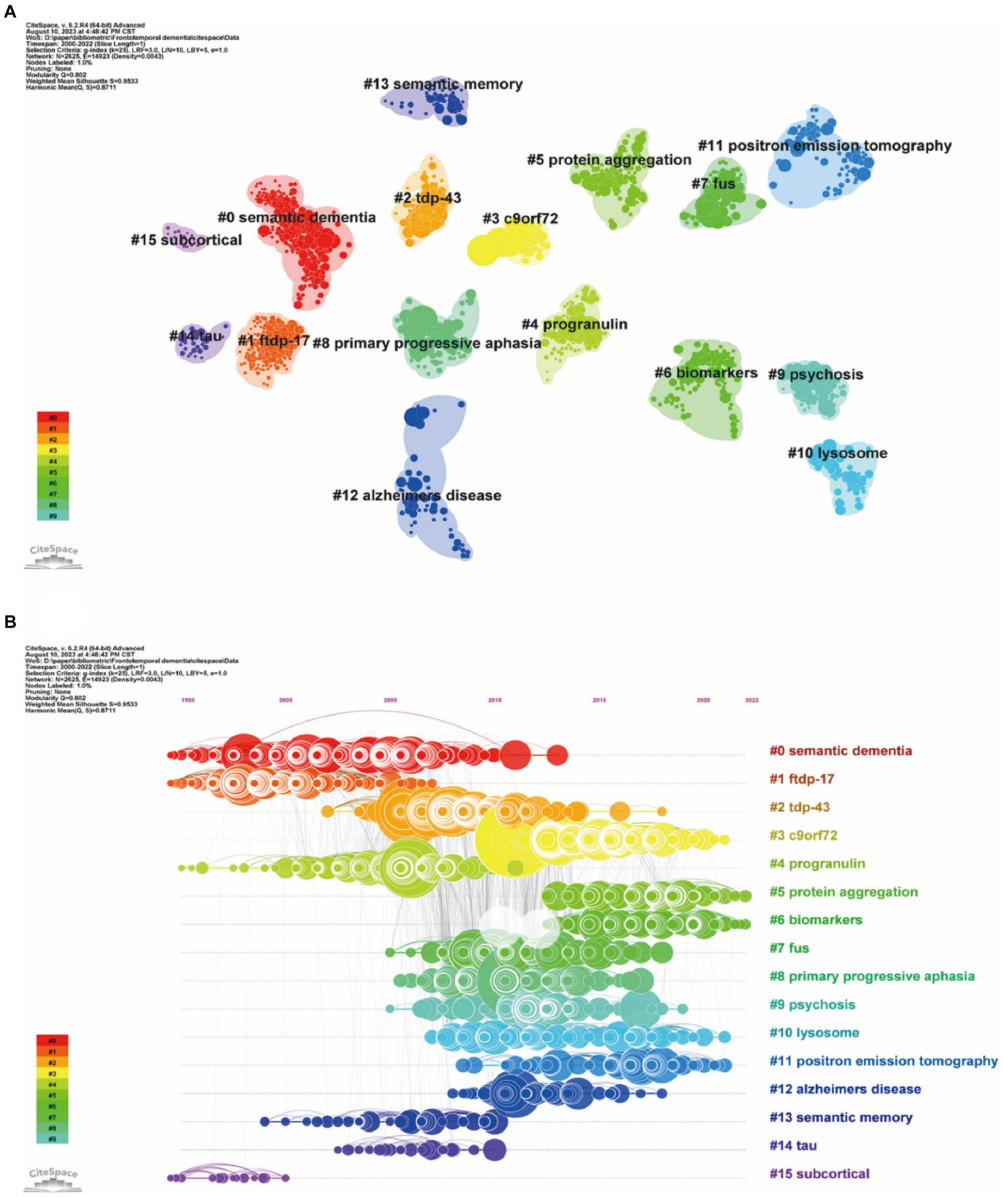
Clustered network map of cocited references **(A)** and their timelines for each cluster label **(B)**. Each node represent one co-cited article, and nodes are organized in different clusters gathered into a network or timeline of cocitation; the node size reflects the cocited counts, and the link indicate the cocited relationship; cluster labels were extracted from keywords.

Clustering of cocited literature is good for finding the cutting edge in a field. The cluster analysis of these cocited references produced 16 main clusters (Figure 7A) and their timelines for each cluster label (Figure 7B). The top 10 clusters that reflect the knowledge base and research progress in FTD are #0 semantic dementia, #1 FTDP-17, #2 TDP-43, #3 C9orF72, #4 progranulin, #5 protein aggregation, #6 biomarkers, #7 FUS, #8 primary progressive aphasia, and #9 psychosis. The modular *Q*-value is 0.80, which is relatively high, indicating that the particularity of scientific mapping has been clearly defined in cocited clustering. In the timeline view of the 16 clusters, the earliest research directions were Cluster #0 semantic dementia, #1 FTDP-17, #4 progranulin, and #15 subcortical, with most papers published approximately 1995. Clusters #3 C9orF72, #5 protein aggregation, #6 biomarkers, #10 lysosome, and #11 positron emission tomography were the main topics of the latest publications, with most papers published approximately 2021.

### 3.6 Analysis of co-occurrence keywords and burst keywords

**Figure 8:**
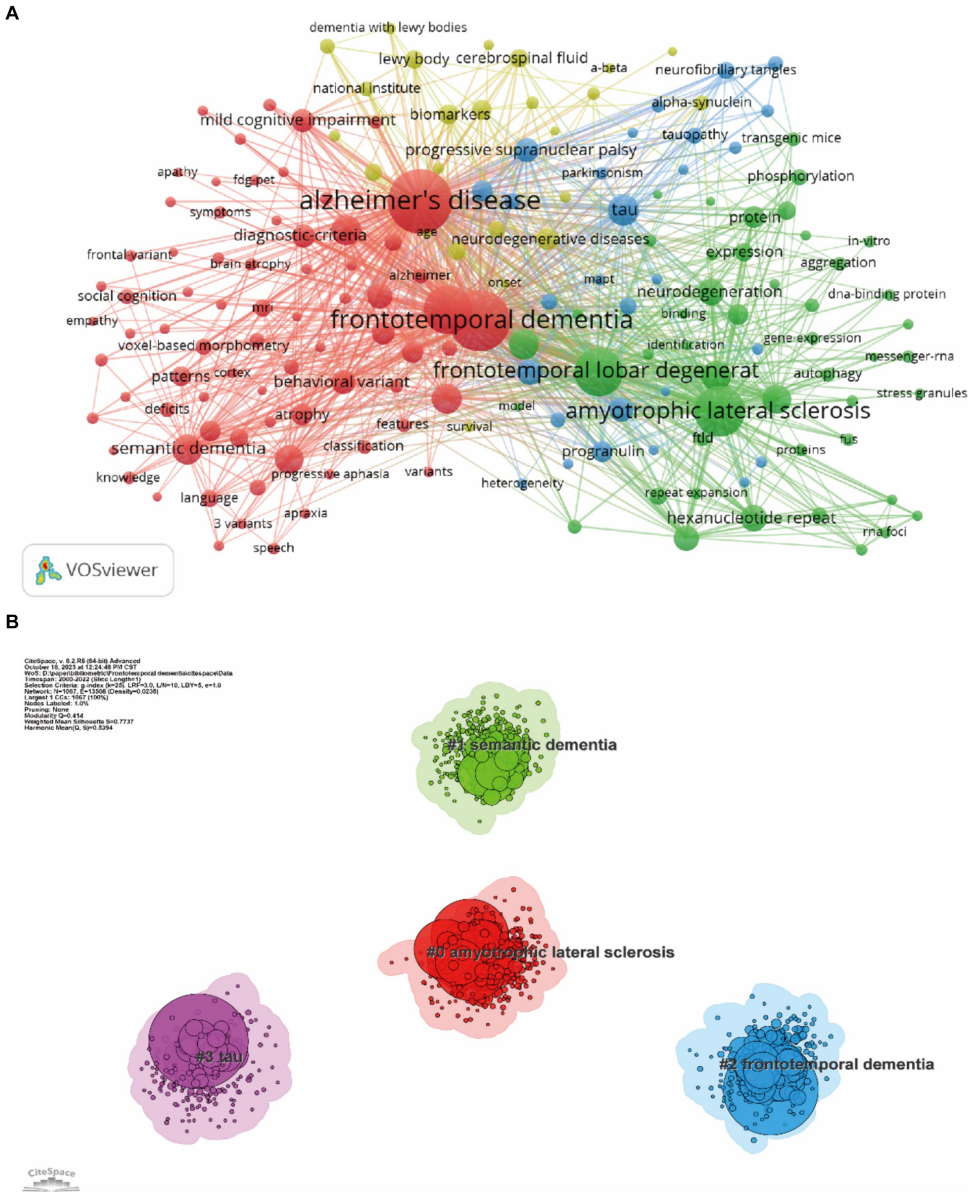
Co-occurrence keywords network **(A)** and the clustered network map of keywords **(B)**. Each node represent one keyword, the size of the circle represents the co-occurrence frequencies, and the link represents the co-occurrence relationship between the two keywords **(A)**. Nodes are organized in different clusters gathered into a network of cocitation, and cluster labels were extracted from keywords **(B)**.

Keyword co-occurrence analysis was used to identify research trends and hotspots in the FTD field. As shown in Table 5, the top 3 high-frequency co-occurrence keywords were “Alzheimer’s disease,” “frontotemporal dementia,” and “amyotrophic lateral sclerosis,” followed by “frontotemporal lobar degeneration,” “dementia,” “tau,” “semantic dementia,” “mutations,” “diagnosis,” and “lobar degeneration.” The co-occurrence keywords network (Figure 8A) and the clustered network map of keywords (Figure 8B) were shown. In this study, keywords were divided into 4 clusters: #0 Amyotrophic lateral sclerosis, #1 semantic dementia, #2 frontotemporal dementia, and #3 tau. When the modularity of the cluster graph is greater than 0.3, the clustering structure is significant, and when the weighted mean silhouette reaches 0.7, the clustering result is convincing. since the modularity was 0.414 and the weighted mean silhouette was 0.77 in this study, the results of the keywords cluster graph can be considered reasonable and convincing. In FTD from 2000 To 2022, the research frontiers were amyotrophic lateral sclerosis, PPA, and tau pathology.

**Table 5:** The top 20 keywords in terms of frequency in frontotemporal dementia research.

Rank	Keyword	Frequency	Rank	Keyword	Frequency
1	Alzheimer’s disease	4,383	11	Disease	947
2	Frontotemporal dementia	3,880	12	TDP-43	917
3	Amyotrophic lateral sclerosis	2,706	13	Primary progressive-aphasia	878
4	Frontotemporal lobar degeneration	2,517	14	Neurodegeneration	733
5	Dementia	2,429	15	Hexanucleotide repeat	727
6	Tau	1,027	16	C9orf72	712
7	Semantic dementia	987	17	Criteria	678
8	Mutations	983	18	Pathology	666
9	Diagnosis	972	19	Progressive supranuclear palsy	661
10	Lobar degeneration	952	20	Behavioral variant	653

**Figure 9:**
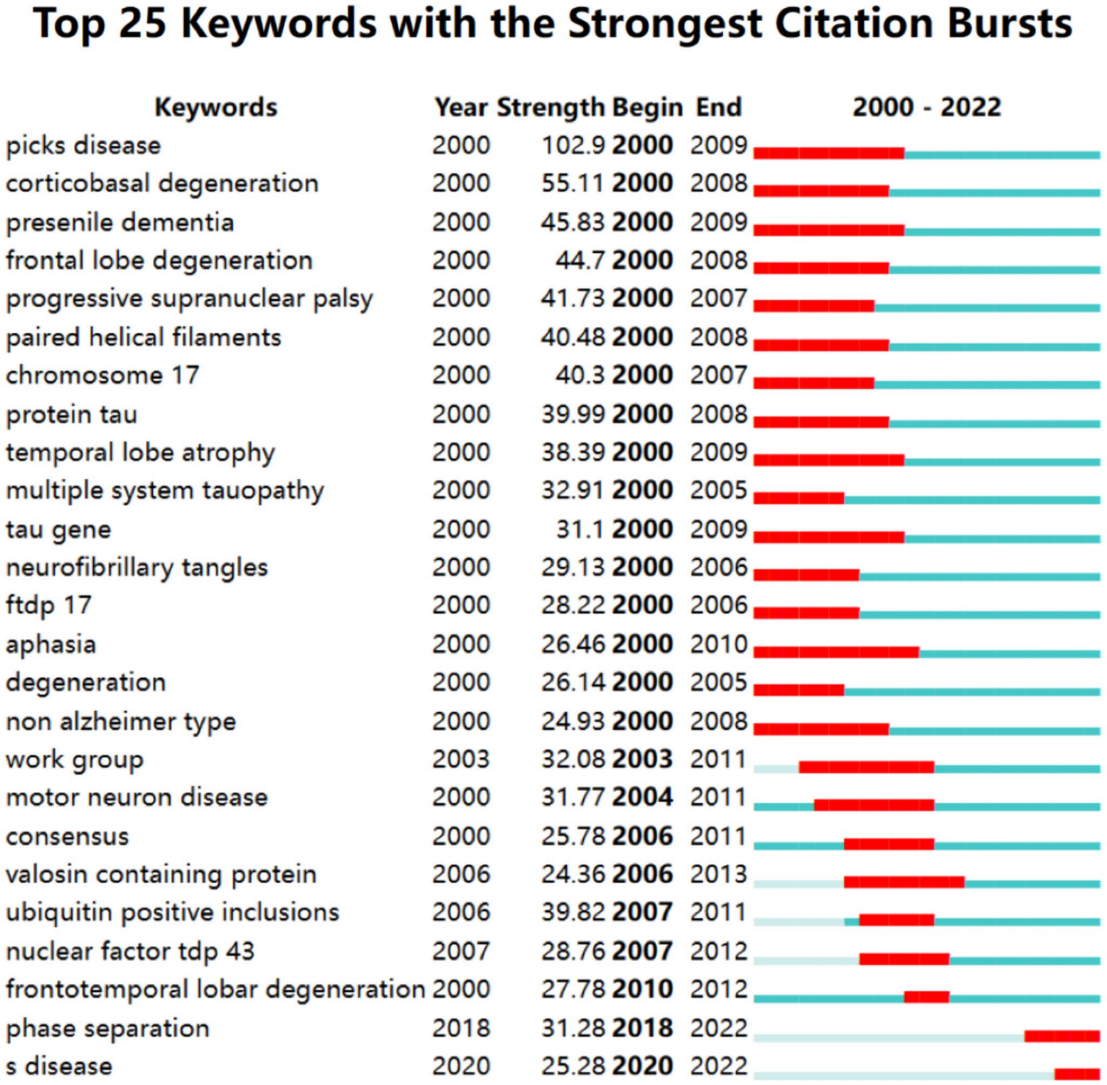
Keywords with the strongest citation bursts in publications on frontotemporal dementia between 2000 and 2022.

Burst keyword detection can identify emerging concepts that have gained significant relevant attention over a period of time. Figure 9 shows the top 25 keywords with the strongest bursts over the past two decades. Picks disease ranked first with the highest burst strength (102.90) followed by corticobasal degeneration (55.11) presenile dementia (45.83) and frontal lobe degeneration (44.70). This indicates that the early understanding of the cerebral cortex characteristics of FTD is in constant progress. From 2000 to 2009 the keywords “tau” “chromosome 17” and “FTDP-17” appeared indicating that the research during this period was related to mutations in the tau gene on chromosome 17. Furthermore phase separation has been the most active burst keyword since 2018 which partly reflects future research trends that may be related to the phase separation of proteins and RNA

## 4 Discussion

### 4.1 General information

Our research found that the annual publications on FTD have increased over the past two decades. The number of published papers in 2022 is 1.76 times that of 2011 and 6.08 times that of 2000. We estimate that the annual number of publications in 2023 is expected to reach 900. FTD is attracting increasing attention and interest from researchers, which may be related to the increased incidence of neurodegenerative diseases caused by population aging (36). Of the top 10 countries/regions contributing to FTD research, the United States, England, and Italy lead the way, accounting for 70%. The top five most productive institutions are California San Francisco, Mayo Clinic, University of Pennsylvania, University College London, and University of Cambridge, which are all from the United States and England. Nodes with high centrality (≥0.10) imply “bridge” effects in the global cooperation network for these countries/regions (37). Among all countries, only England (0.20) and Brazil (0.12) have centrality above 0.1, suggesting that England is likely to maintain a dominant position in FTD research, while Brazil plays an important role in global collaboration. The top three most productive journals are the Journal of Alzheimer’s Disease, Neurobiology of Aging, and Neurology, accounting for 11.3%. This indicates that the researchers focused on neuroscience and geriatrics. Neurosciences, neurology, psychiatry, genetics heredity, and behavioral sciences are the main categories in WOS. In terms of author contributions, Miller, Bruce L. ranks first in publishing papers on FTD (*n* = 427), who focuses on semantic dementia, pathology, Alzheimer’s disease and neuroscience. The 10 most cited papers, mainly related to the diagnostic criteria of FTD, C9orF72, TDP-43, and chromosome 17, were published between 2006 and 2013, which may be a period of high-quality development in the field of FTD research.

### 4.2 Research hotspots and trends in FTD research

Keyword co-occurrence can present Hot topics In an academic field, and keyword bursts indicate emerging trends and potentially valuable research directions to some extent (38). Reference clustering and timelines Can characterize emerging topics in the discipline (16, 39). Based on this, we summarized the research hotspots and trends in FTD research.

#### 4.2.1 Research hotspots

The validation of clinical diagnostic criteria and the subdivision of FTD is one of the research hotspots. The red cluster (Figure 8A) mainly includes the clinical manifestation of FTD according to keywords, such as mild cognitive impairment, semantic dementia, behavior variant, progressive aphasia, and brain atrophy. Typical presentation of FTD includes behavioral and speech variant, forming bvFTD and PPA, respectively. At present, the authoritative diagnostic criteria for bvFTD and PPA still refer to the two consensuses published in 2011 (35, 40). “Possible” bvFTD can be diagnosed by three of six typical manifestations, including disinhibition, apathy, loss of sympathy or empathy, perseverative or compulsive behaviors, hyperorality, and dysexecutive syndrome (35). nfv-PPA was defined as slow, grammatically incorrect language output and difficulty understanding sentences with complex grammar (40). Semantic dementia is characterized by impaired comprehension of naming and individual words, as well as deficits in recognition of object information. Nevertheless, the overlap of symptoms with other syndromes and the occurrence of complications such as other neurodegenerative diseases (Parkinson’s disease or other motor neuron diseases) in the various stages of FTD can still lead to difficulties in the diagnosis and differential diagnosis of FTD (41–43). Subsequently, studies have confirmed the sensitivity and accuracy of these diagnosis criteria (44, 45). For example, the sensitivity of the bvFTD criteria is as high as 85–90% (44). However, with the increasing understanding of FTD, some researchers have proposed that a large proportion of bvFTD and PPA cases are not classified and that the diagnostic criteria are inadequate (46, 47), suggesting that further research to revise the diagnostic criteria and add classifications of FTD are needed. “Alzheimer’s disease,” “Frontotemporal dementia,” “Amyotrophic lateral sclerosis,” “Dementia,” “Semantic dementia,” “Primary progressive-aphasia” are the diseases or syndromes that appear frequently in keywords. The diagnosis and differential diagnosis of FTD from AD, other types of dementia, and ALS have always been the main research content and an important clinical focus (48, 49) because these diseases have similar clinical manifestations and pathological and genetic examinations.

The pathological mechanism of FTD is another important research hotspot. The top 20 keywords included those related to the FTD pathomechanism, such as “Tau,” “Mutations,” “Lobar degeneration,” “Neurodegeneration,” “TDP-43,” “Hexanucleotide repeat,” and “C9orf72.” It has been reported that hyperphosphorylation of MAPT causes FTD (50), presenting tau neurofibrillary inclusion pathology (51). In 2006, Baker et al. (52) demonstrated that mutations in GRN located on chromosome 17q21.31 would cause FTD without mutations in MAPT. Another breakthrough in the same year was that TDP-43 was identified as a major component of sporadic and familial cases of frontotemporal lobar degeneration (FTLD) with ubiquitin-positive, tau-negative inclusions (FTLD-U) and amyotrophic lateral sclerosis (ALS) (53). In 2007, the diagnostic criteria for FTLD were established based on existing criteria and included a new molecular pathology, TDP-43 protein disease, which was considered the most common histological finding in FTLD (54). DeJesus-Hernandez et al. (55) published an article in Neuron in 2011 reporting amplification of the noncoding GGGGCC hexanucleotide repeat of gene C9orF72, which is strongly associated with disease in large relatives of FTD/ALS. This suggested that repeated amplification of C9orF72 was the main cause of FTD and ALS. Additionally, Renton et al. (56) published an article in Neuron in 2011 demonstrating that a massive hexanucleotide repeat expansion within C9orF72 is the cause of chromosome 9p21-linked ALS, FTD and ALS-FTD in 2011. In 2013, Mori et al. (57) found that the C9orf72 GGGGCC repeat is translated into aggregated dipeptide repeat proteins in FTLD/ALS, which are presumably generated by non-ATG-initiated translation from the expanded GGGGCC repeat in three reading frames. These results have made outstanding contributions to the research of FTD and have been published in high-quality journals such as *Nature* (IF = 64.8), *Science* (IF = 56.9), and *Neuron* (IF = 16.2). Further research was being conducted based on the above mechanisms. In addition, “tau” remains the focus of research. As the highest content of microtubule-associated protein, tau protein can bind to the formed microtubules, maintain microtubule stability, and participate in a variety of cellular functions (58). Neurodegenerative diseases associated with abnormal phosphorylation and mutation of tau protein are known as tauopathies, such as frontotemporal lobe degeneration (59). The mechanism of FTD based on pathological changes in tau protein is still unclear. In recent years, researchers have found that it might associate with mitochondrial protein (60), extracellular protein (61), and glutamate signaling (62). The study of the underlying pathogenesis mediated by tau is useful for the targeted blocking treatment of FTD. At present, there is no specific drug for FTD, and the current drug treatment is mainly used to alleviate the symptoms of patients (26). Nonpharmacological treatments have limited benefits to older patients with dementia (63, 64). Therefore, researchers have spent much time studying the pathogenesis of FTD, including pathophysiology, genetics, and biomarkers, in an attempt to explore targeted therapies to treat FTD (65). To date, drugs targeting pathological tau (66) and GRN (67) have been developed.

#### 4.2.2 Emerging trends

It is worth noting that “Phase separation” might be a hotspot starting in 2018 (Figure 9). Protein phase separation (PPS) is widespread in cells and drives a variety of important biological functions (68). PPS at the wrong time or place can create blockages or clumps of molecules associated with neurodegenerative diseases such as Alzheimer’s and Parkinson’s disease (69). This may be an underlying mechanism of FTD. Although much progress has been made in the pathology of FTD, the neuropathology of FTD is heterogeneous (70), and patients with FTD may differ significantly in age of onset, manifestations, and course of disease even with the same mutation (71). Therefore, based on the above biomarkers and pathological changes, researchers have started to diagnose and differentially diagnose FTD from many aspects, such as behavioral manifestations, neuropathology, neuroimaging, and serum markers (72). Focusing on neuropsychological measures of social cognition is of great importance for the early diagnosis of FTD through clinical manifestations (73). Van der Ende et al. (74) measured NPTX2 concentrations in the cerebrospinal fluid of 260 carriers with pathogenic mutations in GRN, C9orf72 or MAPT and some noncarrier individuals and found that NPTX2 might be a meaningful biomarker for the diagnosis of FTD. Zhu et al. (75) found that the binding of plasma glial fibrillary acidic protein and plasma neurofilament light chain could distinguish FTD from AD. Oeckl et al. (76) supported the differential diagnosis of FTD by serum glial fibrillary acidic protein. The pathological changes in the frontotemporal lobe in patients with FTD revealed by multimodal MRI may have certain reference value for the diagnosis and prognosis of FTD (77). Nguyen et al. (78) proposed a novel MRI index called the frontotemporal dementia index, which might help in the differential diagnosis between AD and FTD. In addition, a large-sample longitudinal study on cognitive characteristics and biomarkers of FTD has been reported (79), which has important implications for fully understanding the progression of FTD and diagnosing FTD at different stages. Therefore, the future direction may be biomarker-based cerebrospinal fluid, peripheral blood, and imaging tests and then build predictive models to accurately predict the specific pathological biochemical types of FTD individuals.

### 4.3 Strength and limitations

To our knowledge, this study is the first bibliometric analysis to evaluate the hotspots and cutting edges of FTD-related publications over the past two decades. This study included 10,227 publications on FTD and comprehensively analyzed the number of publications, citations, H-index, collaboration between countries/regions and institutions, cocitations of journals/authors/references, and co-occurrence keywords and burst keywords.

However, there are some limitations in this study. First, we only searched the literature in the SCI-E of the WoSCC database because the articles in WoSCC are the most commonly used data source in bibliometrics and can represent the majority of the information to some extent (40). Second, only English language papers were included, which may ignore relevant studies published in other languages. Third, some recent high-quality publications may not receive enough attention because of low citation frequency, while older articles accumulate more citations. This may diminish the importance of the recently published article. Therefore, readers should be aware that all of this can lead to bias in our results. Finally, bibliometric software cannot distinguish between abbreviations of terms with different names, which can lead to bias in statistical results.

## Data availability statement

The original contributions presented in the study are included in the article/Supplementary material, further inquiries can be directed to the corresponding author.

## Author contributions

XC: Data curation, Formal analysis, Investigation, Methodology, Software, Writing – original draft, Writing – review & editing. YC: Formal analysis, Validation, Writing – original draft, Writing – review & editing. BN: Investigation, Resources, Supervision, Writing – review & editing. CH: Conceptualization, Formal analysis, Funding acquisition, Resources, Writing – review & editing.
