# Supplementary material for: Research trends and hotspots for frontotemporal dementia from 2000 to 2022: a bibliometric analysis
Source: Front Neurol. 2024 Jul 17;15:1399600. doi: 10.3389/fneur.2024.1399600 (PMC11288951; doi:10.3389/fneur.2024.1399600)
Supplement: Supplementary file 1 [file Data_Sheet_1.docx]

Web of Science

((TI=("Frontotemporal Dementia*" OR “Dementia*, Frontotemporal” OR “Multiple System Tauopathy with Presenile Dementia” OR “Dementia*, Ubiquitin-Positive Frontotemporal” OR “Dementia, Hereditary Dysphasic Disinhibition” OR “Frontotemporal Lobe Dementia*” OR “Lobe Dementia*, Frontotemporal” OR “Wilhelmsen-Lynch Disease*” OR “Disease*, Wilhelmsen-Lynch” OR “Frontotemporal Lobar Degeneration” OR “Disinhibition-Dementia-Parkinsonism-Amyotrophy Compl*” OR “Disinhibition Dementia Parkinsonism Amyotrophy Complex” OR “Compl*, Disinhibition-Dementia-Parkinsonism-Amyotrophy” OR “Disinhibition-Dementia-Parkinsonism-Amytrophy Compl*” OR “Compl*, Disinhibition-Dementia-Parkinsonism-Amytrophy” OR “Disinhibition Dementia Parkinsonism Amytrophy Complex” OR “Familial Pick* Disease*” OR “Disease*, Familial Pick’s” OR “Pick’s Disease*, Familial” OR “Hereditary Dysphasic Disinhibition Dementia” OR “FTDP-17” OR “DDPAC” OR “GRN-Related Frontotemporal Dementia” OR “Dementia*, GRN-Related Frontotemporal” OR “FTD- GRN” OR “FTD-PGRN” OR “FTLD-17 GRN” OR “FTLD with TDP-43 Pathology” OR “FTLD with TDP 43 Pathology” OR “FTLD-TDP” OR “HDDD1” OR “HDDD2” OR “Semantic Dementia*” OR “Dementia*, Semantic” OR “primary progressive aphasia” OR “progressive nonfluent aphasia” OR “progressive non-fluent aphasia” OR “frontolobar degeneration” OR “frontal lobar degeneration”)) OR AB=("Frontotemporal Dementia*" OR “Dementia*, Frontotemporal” OR “Multiple System Tauopathy with Presenile Dementia” OR “Dementia*, Ubiquitin-Positive Frontotemporal” OR “Dementia, Hereditary Dysphasic Disinhibition” OR “Frontotemporal Lobe Dementia*” OR “Lobe Dementia*, Frontotemporal” OR “Wilhelmsen-Lynch Disease*” OR “Disease*, Wilhelmsen-Lynch” OR “Frontotemporal Lobar Degeneration” OR “Disinhibition-Dementia-Parkinsonism-Amyotrophy Compl*” OR “Disinhibition Dementia Parkinsonism Amyotrophy Complex” OR “Compl*, Disinhibition-Dementia-Parkinsonism-Amyotrophy” OR “Disinhibition-Dementia-Parkinsonism-Amytrophy Compl*” OR “Compl*, Disinhibition-Dementia-Parkinsonism-Amytrophy” OR “Disinhibition Dementia Parkinsonism Amytrophy Complex” OR “Familial Pick* Disease*” OR “Disease*, Familial Pick’s” OR “Pick’s Disease*, Familial” OR “Hereditary Dysphasic Disinhibition Dementia” OR “FTDP-17” OR “DDPAC” OR “GRN-Related Frontotemporal Dementia” OR “Dementia*, GRN-Related Frontotemporal” OR “FTD- GRN” OR “FTD-PGRN” OR “FTLD-17 GRN” OR “FTLD with TDP-43 Pathology” OR “FTLD with TDP 43 Pathology” OR “FTLD-TDP” OR “HDDD1” OR “HDDD2” OR “Semantic Dementia*” OR “Dementia*, Semantic” OR “primary progressive aphasia” OR “progressive nonfluent aphasia” OR “progressive non-fluent aphasia” OR “frontolobar degeneration” OR “frontal lobar degeneration”)) OR AK=("Frontotemporal Dementia*" OR “Dementia*, Frontotemporal” OR “Multiple System Tauopathy with Presenile Dementia” OR “Dementia*, Ubiquitin-Positive Frontotemporal” OR “Dementia, Hereditary Dysphasic Disinhibition” OR “Frontotemporal Lobe Dementia*” OR “Lobe Dementia*, Frontotemporal” OR “Wilhelmsen-Lynch Disease*” OR “Disease*, Wilhelmsen-Lynch” OR “Frontotemporal Lobar Degeneration” OR “Disinhibition-Dementia-Parkinsonism-Amyotrophy Compl*” OR “Disinhibition Dementia Parkinsonism Amyotrophy Complex” OR “Compl*, Disinhibition-Dementia-Parkinsonism-Amyotrophy” OR “Disinhibition-Dementia-Parkinsonism-Amytrophy Compl*” OR “Compl*, Disinhibition-Dementia-Parkinsonism-Amytrophy” OR “Disinhibition Dementia Parkinsonism Amytrophy Complex” OR “Familial Pick* Disease*” OR “Disease*, Familial Pick’s” OR “Pick’s Disease*, Familial” OR “Hereditary Dysphasic Disinhibition Dementia” OR “FTDP-17” OR “DDPAC” OR “GRN-Related Frontotemporal Dementia” OR “Dementia*, GRN-Related Frontotemporal” OR “FTD- GRN” OR “FTD-PGRN” OR “FTLD-17 GRN” OR “FTLD with TDP-43 Pathology” OR “FTLD with TDP 43 Pathology” OR “FTLD-TDP” OR “HDDD1” OR “HDDD2” OR “Semantic Dementia*” OR “Dementia*, Semantic” OR “primary progressive aphasia” OR “progressive nonfluent aphasia” OR “progressive non-fluent aphasia” OR “frontolobar degeneration” OR “frontal lobar degeneration”)
